# ﻿The genus *Pseudophanias* Raffray (Coleoptera, Staphylinidae, Pselaphinae) from Nanling Priority Area for Biodiversity Conservation, China

**DOI:** 10.3897/zookeys.1179.110478

**Published:** 2023-09-12

**Authors:** Yong-Qin Zhang, Zi-Wei Yin

**Affiliations:** 1 Laboratory of Systematic Entomology, College of Life Sciences, Shanghai Normal University, Shanghai 200234, China Shanghai Normal University Shanghai China

**Keywords:** Ant-loving beetle, Guangxi, Guizhou, new species, Oriental region, Tmesiphorini

## Abstract

Prior to this study, no species of *Pseudophanias* Raffray had been reported from Nanling, a vast biodiversity conservation area that spans five provinces in southern China. In this paper, three new species of the genus are described: *Pseudophaniasfurcilobus***sp. nov.** (Guizhou, Guangxi), *P.leigong***sp. nov.** (Guizhou), and *P.mulun***sp. nov.** (Guangxi), suggesting that additional study on the diversity of this group in the area is required. These species are characterized, keyed, and compared to similar congeners, supplemented with illustrations of the habitus and other morphological characters.

## ﻿Introduction

After a recent discovery of a morphologically unusual representative at West Tianmu Mountain, East China (Yin and Zhao 2022), the Oriental ant-loving beetle genus *Pseudophanias* Raffray (Pselaphitae: Tmesiphorini) now includes 20 species scattered throughout Malaysia (5 spp.), Indonesia (4 spp.), Singapore (2 spp.), Thailand (1 sp.), Myanmar (2 spp.), Japan (2 spp.), Nepal (1 sp.) and China (4 spp.). While a majority of specimens of these species were collected in leaf-litter layer, a few are probably myrmecophilous (e.g., Yin 2019), or cavernicolous (Yin et al. 2015). The group is diverse in tropical areas of Southeast Asia, with numerous new species having been seen in various museum collections (Yin pers. obs.).

The four species of China were all recently described. Two of them inhabit Taiwan (Inoue et al. 2020), and one each in Yunnan (Yin 2019) and Zhejiang (Yin and Zhao 2022), but information on the diversity of the genus in southern China remains virtually absent. As a part of an ongoing project documenting the Pselaphinae fauna of Nanling, which was recently proposed as a vast biodiversity conservation belt spanning Jiangxi, Hunan, Guangdong, Guangxi, and Guizhou provinces (https://www.mee.gov.cn/gkml/hbb/bgg/201601/t20160105_321061.htm), we report the first occurrence of *Pseudophanias* and describe three new species. The limited available specimens, both quantitatively and geographically, suggests that additional members of the genus are likely to be found from suitable forest habitats in the region.

## ﻿Material and method

The material treated in this paper is deposited in the Insect Collection of Shanghai Normal University (**SNUC**). The label data of the material are quoted verbatim.

Dissected parts were mounted in Euparal on plastic slides pinned with the specimen. The habitus image of the beetle was taken using a Canon EOS R5 camera, equipped with a 7.5× Mitutoyo M Plan Apo lens, and two Godox V860III-C TTL Li-Ion flashes were used as the light source. Images of morphological details were produced using a Canon G9 camera mounted to an Olympus CX31 microscope under reflected or transmitted light. Zerene Stacker v. 1.04 was used for image stacking. All images were modified and grouped into plates using Adobe Photoshop CC 2020.

Measurements were taken as follows: total body length was measured from the anterior margin of the rostrum to the apex of the abdomen; head length was measured from the anterior margin of the rostrum to the head base, excluding the cervical constriction; head width was measured across the eyes; the length of the pronotum was measured along the midline, the width of the pronotum equals the maximum width; the length of the elytra was measured along the suture; the width of the elytra was measured as the maximum width across both elytra; the length of the abdomen is the length of the dorsally exposed part of the abdomen along its midline, the width is the maximum width.

The terminology follows Chandler (2001) and Yin (2022). Abdominal tergites and sternites are numbered in Arabic (starting from the first visible segment) and Roman (reflecting true morphological position) numerals, e.g., tergite 1 (IV), or sternite 1 (III). Paired appendages in the description are treated as singular.

## ﻿Taxonomy

### ﻿ Pseudophanias furcilobus

Taxon classificationAnimaliaColeopteraStaphylinidae

Y.-Q. Zhang, Z.-W. Yin sp. nov.

7C1E838D-58E1-5F6A-B860-6C3A46A6EE0B

https://zoobank.org/7E7AF0E7-3D34-489D-BA4F-982AC8A097F6

**Figure 1.**
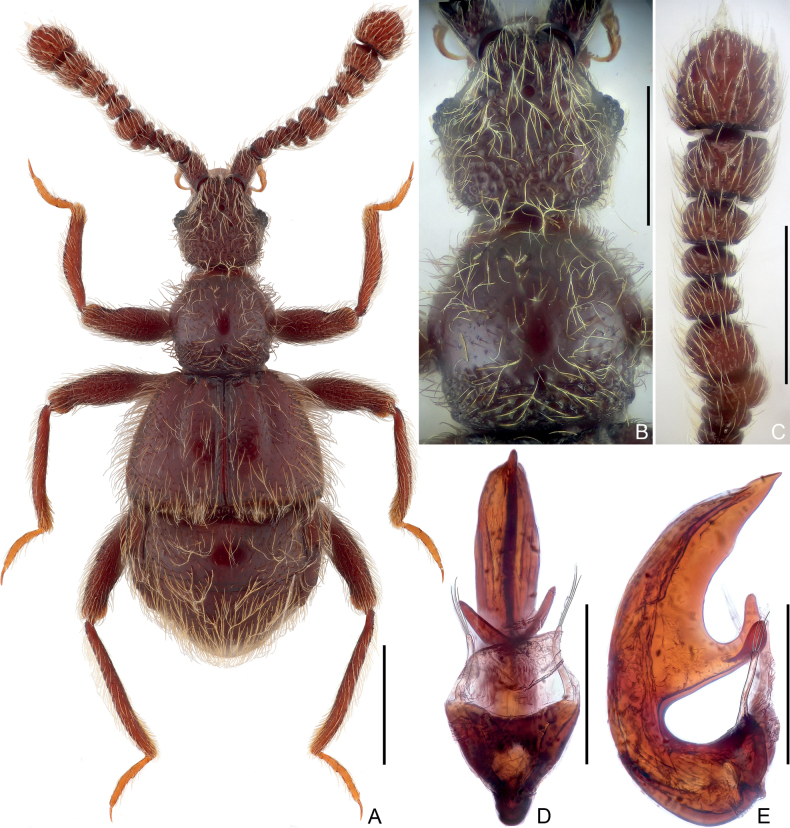
Habitus and morphological details of *Pseudophaniasfurcilobus* sp. nov., male **A** dorsal habitus **B** head and pronotum **C** antennal club **D, E** aedeagus, ventral (**D**), and lateral (**E**). Scale bars: 0.5 mm (**A**); 0.3 mm (**B, C**); 0.2 mm (**D, E**).

Figs 1 , 4A Chinese common name: 叉茎隐须蚁甲

#### Type material

**(4 exx.). *Holotype*: China**: ♂: ‘China: Guizhou, Libo County, Maolan N. R., 25°16′52″N, 107°54′18″E, 850–890 m, 20.vii.2015, Chen & Zhao leg. (贵州荔波茂兰自然保护区)’ (SNUC). ***Paratypes*: China**: 1 ♀, same collecting data as for holotype; 1 ♂, 1 ♀, ‘China: Guangxi, Hechi City, Mulun N. R., 25°3′12″N, 107°57′59″E, 450–600 m, 26.vii.2015, Chen, He & Hu leg. (广西河池木论自然保护区)’ (SNUC).

#### Diagnosis.

**Male.** Body moderately elongate, length approximately 2.1 mm. Vertex coarsely punctate, with punctiform vertexal and slightly larger frontal fovea. Antennomeres 5–11 modified, each distinctly transverse, antennomeres 5 as broad as 6, 7 and 8 much narrower than 6, 9 and 10 successively larger, 11 largest, semiglobular, truncate at base. Pronotum with finely punctate disc, coarsely punctate at basal part. Legs simple. Tergite 1 (IV) about 2.4 times as long as 2 (V), lacking discal carinae. Median lobe of aedeagus at middle greatly projected ventrally, projection deeply forked apically; parameres each with three long setae at apex. **Female.** Similar to male in external morphology; antenna lacking modifications.

#### Description.

**Male. *Body*** (Fig. 1A) length 2.10–2.12 mm; color reddish-brown, tarsi and mouthparts lighter. Dorsal surface finely punctate, covered with long pubescence.

***Head*** (Fig. 1B) much longer than wide, length 0.43–0.45 mm, width across eyes 0.41–0.42 mm, sub-hexagonal; vertex coarsely punctate, with narrowly separated, punctiform foveae (dorsal tentorial pits); tempora much longer than eyes; moderately convergent posteriorly; frons broadly and shallowly impressed at middle, with small frontal fovea, rostrum approximately half as wide as head, clypeus sharply descending, smooth, anterior margin carinate and raised, setose C-shaped sulci broad, clearly visible in dorsal view. Eyes moderately prominent, each composed of approximately 30 ommatidia. Maxillary palpi symmetric, palpomere 1 minute, 2 elongate, curved, lengthily pedunculate in basal 2/3, 3 short, sub-trapezoidal, widest at apex, 4 fusiform, widest before base. Antenna elongate, length 0.99–1.01 mm; club formed by enlarged antennomeres 5–11 (Fig. 1C); antennomere 1 approximately as long as 2 and 3 combined, subcylindrical, 2–4 each short, 5 and 6 greatly transverse, subequal in width, 6 along axis twice length of 5, 7 to 9 narrower and shorter than 6, 8 shortest, 10 much larger than 9, transversely subquadrate, 11 largest, semiglobular, truncate at base.

***Pronotum*** (Fig. 1B) slightly wider than long, length 0.42–0.44 mm, width 0.45–0.46 mm, widest at middle; lateral margins rounded, convergent anteriorly and posteriorly; anterior margin smoothly curved, posterior margin evenly convex posteriorly; disc moderately convex, finely punctate, with tiny, asetose median and lateral antebasal foveae, basal collar roughly punctate. Prosternum with anterior part slightly shorter than coxal part, with small lateral procoxal foveae; hypomera fused with sternum, smooth; margin of coxal cavity non-carinate.

***Elytra*** much wider than long, length 0.51–0.54 mm, width 0.85–0.86 mm; roundly trapezoidal, dorsal surface with dense, long pubescence; each elytron with two large, asetose, basal foveae; discal striae shallow and wide, extending from outer basal foveae posteriorly for 5.6/10 elytral length. Humeral denticles absent, humeri almost flat, lacking subhumeral foveae or marginal striae; posterolateral margins shortly oblique with row of dense setae. Metathoracic wings greatly reduced, short.

***Mesoventrite*** short, laterally fused with metaventrite; median mesoventral foveae widely separated, originating from shared setose, transverse opening, large lateral mesoventral foveae unforked internally, with short, apically truncate mesoventral process. Metaventrite (Fig. 4A) finely punctate, convex medially, area anterior to posterior margin roundly and shallowly impressed at middle; posterior margin at middle roundly and moderately deeply emarginate.

All legs elongate and slender; femora coarsely punctate; each tarsus with one major and one reduced setiform pretarsal claw.

***Abdomen*** widest at lateral margins of tergite 1 (IV), length 0.71– 0.72 mm, width 0.84–0.85 mm. Tergite 1 longest, approximately 2.4× as long as 2 (V), with broad, setose basal sulcus and pair of basolateral foveae, lacking discal carinae; tergites 2–4 (V–VII) each lacking basal sulcus or fovea, 2 and 3 successively shorter, 4 distinctly longer than 2 and 3; tergite 5 (VIII) transverse, posterior margin evenly rounded. Sternite 2 (IV) at middle slightly longer than 3–5 (V–VII) combined, with deep, setose basal impression and pair of basolateral sockets, 3 and 4 each short at middle, combined longer than 5 (VII), 3–5 each lacking sulcus or fovea at base, 6 (VIII) transverse, posterior margin slightly convex at middle.

***Aedeagus*** (Fig. 1D, E) 0.46 mm in length, moderately sclerotized, dorso-ventrally almost symmetric; median lobe strongly curved in lateral view, at middle greatly projected ventrally, projection deeply forked apically, parameres elongate, each with three long, curved setae at apex.

**Female.** General external morphology similar to male; antenna slightly shorter, unmodified; each eye composed of about 30 ommatidia; humeral flat; metathoracic wings similar to male, reduced. Measurements (as for male): body length 2.09–2.11 mm, length/width of head 0.44–0.46 mm/0.39–0.40 mm, length/width of pronotum 0.43–0.44 mm/0.43–0.44 mm, length/width of elytra 0.48–0.49 mm/0.83–0.88 mm, length/width of abdomen 0.74–0.82 mm/0.83–0.89 mm.

#### Comparative notes.

The male of this species is morphologically similar to *Pseudophaniasexcavatus* Inoue, Nomura & Yin from Taiwan, China by the antennal modification composed of apical seven antennomeres (Inoue et al. 2020), i.e., antennomeres 5–11 greatly transverse and distinctly larger than 1–4. They can be readily separated by the different shapes of the antennal modification and aedeagus. In *P.excavatus* the antennomeres 5 and 10 have their ventral surfaces greatly projected (Inoue et al. 2020: fig. 10B) (projection lacking in *P.furcilobus* sp. nov.), and the median lobe of the aedeagus lacks a ventral projection, and the apical part in lateral view is greatly extended, twisted, and more strongly recurved (Inoue et al. 2020: fig. 13B).

#### Distribution.

Southwestern China: Guizhou, Guangxi.

#### Etymology.

The specific epithet is a combination of Latin stem *furc*, meaning, “fork”, and noun *lobus*, meaning, “lobe”, referring to the apically forked ventral projection of median lobe of the aedeagus.

### ﻿ Pseudophanias leigong

Taxon classificationAnimaliaColeopteraStaphylinidae

Y.-Q. Zhang, Z.-W. Yin sp. nov.

317D7F30-A515-5FFD-A9E4-4AC7A95E1102

https://zoobank.org/0A86E774-3A44-4849-AAB4-C20DC0E4AB31

**Figure 2.**
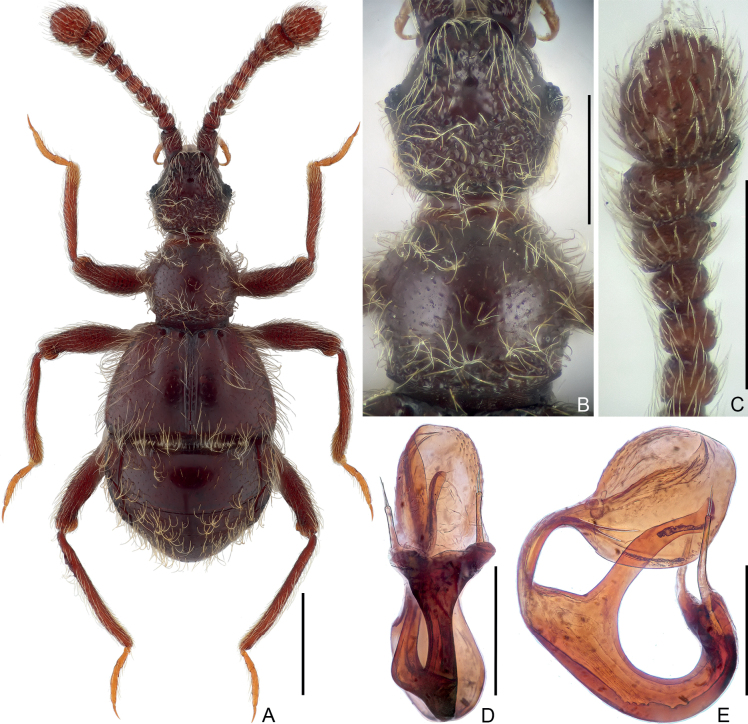
Habitus and morphological details of *Pseudophaniasleigong* sp. nov., male **A** dorsal habitus **B** head and pronotum **C** antennal club **D, E** aedeagus, ventral (**D**), and lateral (**E**). Scale bars: 0.5 mm (**A**); 0.3 mm (**B, C**); 0.2 mm (**D, E**).

Figs 2 , 4B Chinese common name: 雷公隐须蚁甲

#### Type material

**(1 ex.). *Holotype*: China**: ♂: ‘China: Guizhou, Leishan, Leigong Mt, Xiannütang., 26°22'22.11"N, 108°11'52.12"E, 1550 m, 3.v.2021, Tang, Peng, Cai, Song leg. (贵州雷公县雷公山仙女塘)’ (SNUC).

#### Diagnosis.

**Male.** Body moderately elongate, length 2.0 mm. Vertex coarsely punctate, with punctiform vertexal and frontal fovea. Antennomeres 9–11 enlarged, successively larger, much wider than other antennomeres, 10 ventrally protruding on apical margin, 11 hemispherical, truncate at base. Pronotum with smooth disc, coarsely punctate at basal part. Legs simple. Tergite 1 (IV) more than 2.5 times as long as 2 (V), lacking discal carinae. Median lobe of aedeagus in lateral view strongly curved, at middle greatly projected ventrally, and markedly enlarged at apex; parameres each with two long setae at apex. **Female.** Unknown.

#### Description.

**Male. *Body*** (Fig. 2A) length 2.0 mm; color reddish-brown, tarsi and mouthparts lighter. Dorsal surface finely punctate, covered with dense pubescence.

***Head*** (Fig. 2B) approximately as long as wide, length 0.46 mm, width across eyes 0.45 mm, sub-hexagonal; vertex coarsely punctate, with narrowly separated, punctiform foveae (dorsal tentorial pits); tempora much longer than eyes, moderately convergent posteriorly; frons longitudinally and shallowly impressed at middle, with punctiform frontal fovea, rostrum approximately half as wide as head; clypeus sharply descending, with anterior margin moderately carinate and raised, setose C-shaped sulci surrounding antennal insertions clearly visible in dorsal view. Eyes weakly prominent, each composed of approximately 18 ommatidia. Maxillary palpi symmetric, palpomere 1 minute, 2 elongate, curved, lengthily pedunculate in basal 2/3, 3 short, sub-trapezoidal, widest at apex, 4 fusiform, widest before base. Antenna elongate, length 0.92 mm; club formed by enlarged apical three antennomeres 9–11 (Fig. 2C); antennomere 1 thick and elongate, subcylindrical, 2–8 each sub-moniliform, 2, 3, 4, 5, 8 distinctly transverse, 6 and 7 subquadrate, 8 shortest, 9 much larger than 8, 10 much wider and longer than 9, angularly protruding ventrally on apical margin, 11 suboval, largest, truncate at base.

***Pronotum*** (Fig. 2B) much wider than long, length 0.43 mm, width 0.45 mm, widest at middle; lateral margins rounded, convergent anteriorly and posteriorly; anterior margin slightly and smoothly curved, posterior margin evenly convex posteriorly; disc moderately convex, finely punctate, with tiny, asetose median and lateral antebasal foveae, basal collar roughly punctate. Prosternum at middle with anterior part slightly longer than coxal part, with small, broadly separated lateral procoxal foveae; hypomera fused with sternum, smooth; margin of coxal cavity non-carinate.

***Elytra*** much wider than long, length 0.48 mm, width 0.84 mm; roundly trapezoidal, dorsal surface with long pubescence; each elytron with two large, asetose, basal foveae; discal striae shallow and wide, extending from outer basal foveae to apical 5.4/10 of elytral length. Humeral denticles absent, humeri almost flat, lacking subhumeral foveae or marginal striae; posterolateral margins shortly oblique. Metathoracic wings absent.

***Mesoventrite*** short, laterally fused with metaventrite; median mesoventral foveae widely separated, originating from shared setose, transverse opening, large lateral mesoventral foveae unforked internally, with short, apically roundly truncate mesoventral process. Metaventrite (Fig. 4B) strongly convex, area anterior to posterior margin roundly impressed at middle; posterior margin at middle roundly and moderately deeply emarginate.

All legs elongate and slender; femora coarsely punctate; each tarsus with one major and one reduced setiform pretarsal claw.

***Legs*** elongate; femora coarsely punctate; each tarsus with one major and one reduced setiform pretarsal claw.

***Abdomen*** widest at lateral margins of tergite 1 (IV), length 0.67 mm, width 0.83 mm. Tergite 1 longest, more than 2.5× as long as 2 (V), with broad, setose basal sulcus and pair of basolateral foveae, lacking discal carinae; tergites 2–4 (V–VII) each lacking basal sulcus or fovea, 2 and 3 successively shorter, 4 distinctly longer than 2 and 3; tergite 5 (VIII) semicircular, transverse, posterior margin evenly rounded. Sternite 2 (IV) at middle approximately as long as 3–5 (V–VII) combined, with densely setose basal sulcus and pair of basolateral foveae at lateral ends of sulcus, 3–5 each short at middle, lacking sulcus or fovea at base, 6 (VIII) transverse, posterior margin slightly convex at middle.

***Aedeagus*** (Fig. 2D, E) 0.46 mm in length, well sclerotized, dorso-ventrally slightly asymmetric; median lobe strongly curved in lateral view, at middle greatly extended ventrally to form elongate projection, apex markedly enlarged, globular in lateral view; parameres elongate, each with two long apical setae.

**Female.** Unknown.

#### Comparative notes.

The male of this species is externally similar to those of *P.furcilobus* and *P.mulun*, both described here, but can be readily separated by the different antennal modification, i.e., only antennomeres 9–11 enlarged in *P.leigong* sp. nov. vs. antennomeres 6–11 enlarged in the latter two species, and the configuration of the aedeagus.

#### Distribution.

Southwestern China: Guizhou.

#### Etymology.

The named is taken from Leigong Mountain, the type locality of the new species.

### ﻿ Pseudophanias mulun

Taxon classificationAnimaliaColeopteraStaphylinidae

Y.-Q. Zhang, Z.-W. Yin sp. nov.

8C20A0A0-8667-5D23-9414-92686F55EBE6

https://zoobank.org/AB75AACF-0220-45E1-BE38-6C4E7CE21983

**Figure 3.**
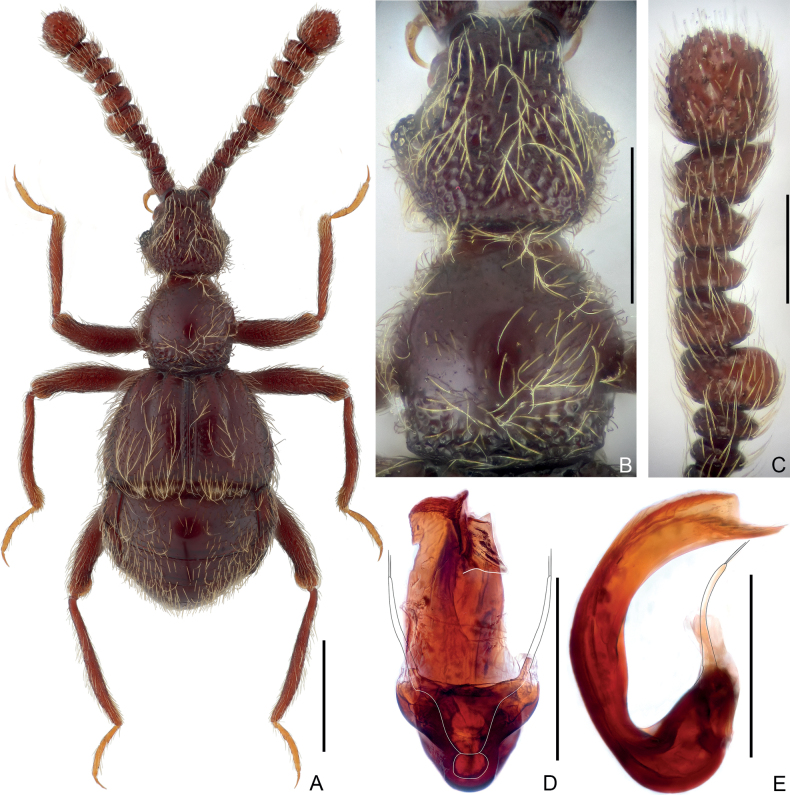
Habitus and morphological details of *Pseudophaniasmulun* sp. nov., male **A** dorsal habitus **B** head and pronotum **C** antennal club **D, E** aedeagus, ventral (**D**), and lateral (**E**). Scale bars: 0.5 mm (**A**); 0.3 mm (**B, C**); 0.2 mm (**D, E**).

**Figure 4.**
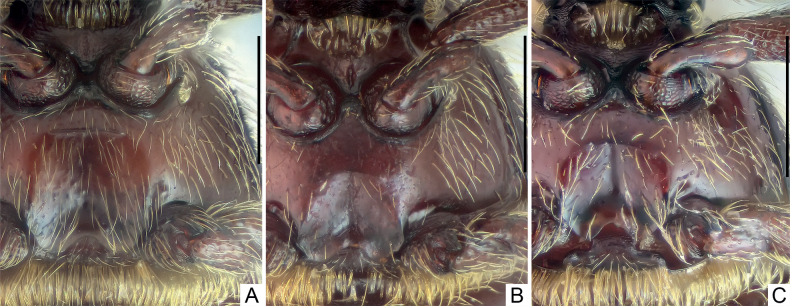
Male metaventrite **A***Pseudophaniasfurcilobus***B***P.leigong***C***P.mulun*. Scale bars: 0.3 mm.

Figs 3 , 4C Chinese common name: 木论隐须蚁甲

#### Type material

**(1 ex.). *Holotype*: China**: ♂: ‘China: Guangxi, Hechi City, Mulun N. R., 25°12'14"N, 108°5'46"E (at the broader of Guangxi and Guizhou), 460 m, 27.vii.2015, Chen, He & Hu leg. (广西河池木论自然保护区)’ (SNUC).

#### Diagnosis.

**Male.** Body moderately elongate, length approximately 1.9 mm. Vertex coarsely punctate, with punctiform vertexal and frontal fovea. Antennomeres 6–11 enlarged, each distinctly transverse, antennomere 69 much wider than 7–9 and as wide as 10, 10 angularly protruding ventrally. Pronotum with smooth disc, coarsely punctate at basal part. Legs simple. Tergite 1 (IV) more than 2.5 times as long as 2 (V), lacking discal carinae. Median lobe of aedeagus in lateral view C-shaped, abruptly narrowed before apex; parameres each with two long setae at apex.

#### Description.

**Male. *Body*** (Fig. 3A) length 1.89 mm; color reddish-brown, tarsi and mouthparts lighter. Dorsal surface finely punctate, covered with dense pubescence. **Female.** Unknown.

***Head*** (Fig. 3B) approximately as long as wide, length 0.4 mm, width across eyes 0.41 mm, sub-hexagonal; vertex coarsely punctate, with narrowly separated, punctiform foveae (dorsal tentorial pits); tempora much longer than eyes, convergent posteriorly; frons longitudinally and shallowly impressed at middle, with punctiform frontal fovea, rostrum approximately half as wide as head; clypeus sharply descending, with anterior margin moderately carinate and raised; setose C-shaped sulci broad, clearly visible in dorsal view. Eyes weakly prominent, each composed of approximately 16 ommatidia. Maxillary palpi symmetric, palpomere 1 minute, 2 elongate, curved, lengthily pedunculate in basal 2/3, 3 short, sub-trapezoidal, widest at apex, 4 fusiform, widest before base. Antenna elongate, length 0.95 mm; antennomere 1 approximately as long as 2 and 3 combined, 2 much shorter than 1, moderately transverse, 3–5 each short, distinctly transverse, 6–11 (Fig. 3C) enlarged, 6, 10 and 11, and 7–9 subequal in maximum width, respectively, 10 angularly protruding ventrally, 11 suboval, largest, truncate at base.

***Pronotum*** (Fig. 3B) slightly wider than long, length 0.40 mm, width 0.42 mm, widest at middle; lateral margins rounded, convergent anteriorly and posteriorly; anterior margin slightly and smoothly curved, posterior margin evenly convex posteriorly; disc weakly convex, finely punctate, with tiny median and small lateral antebasal foveae, basal collar roughly punctate. Prosternum at middle with anterior part slightly longer than coxal part, with small, broadly separated lateral procoxal foveae; hypomera fused with prosternum; margins of coxal cavities non-carinate.

***Elytra*** much wider than long, length 0.49 mm, width 0.77 mm; roundly trapezoidal, dorsal surface with dense, long pubescence; each elytron with two large, asetose, basal foveae; discal striae broad and shallow, extending from outer basal foveae posteriorly for 5.4/10 elytral length; humeral denticles absent, humeri weakly protuberant, lacking subhumeral foveae or marginal striae; posterolateral margins shortly oblique. Metathoracic wings absent.

***Mesoventrite*** short, laterally fused with metaventrite; median mesoventral foveae widely separated, originating from shared setose, transverse opening, large lateral mesoventral foveae unforked internally, with short, apically truncate mesoventral process. Metaventrite (Fig. 4C) strongly convex, area anterior to posterior margin roundly impressed at middle; posterior margin at middle roundly and deeply emarginate.

***Legs*** elongate and slender; femora coarsely punctate; each tarsus with one major and one reduced setiform pretarsal claw.

***Abdomen*** widest at lateral margins of tergite 1 (IV), length 0.67 mm, width 0.76 mm. Tergite 1 (IV) longest, more than 2.5× as long as 2 (V), with broad, setose basal sulcus and pair of basolateral foveae, lacking discal carinae; tergite 2 (V) slightly longer than 3 (VI), 4 (VII) distinctly longer than 2 and 3, 2–4 each lacking basal sulcus or fovea; tergite 5 (VIII) semicircular, transverse, posterior margin evenly rounded. Sternite 2 (IV) at middle approximately as long as 3–5 (V–VII) combined, with deep, setose basal impression and pair of basolateral sockets, 3 (V) and 4 (VI) each short at middle, combined approximately as long as 5 (VII), 3–5 lacking sulcus or fovea at base, 6 (VIII) transverse, posterior margin slightly convex at middle.

***Aedeagus*** (Fig. 3D, E) 0.33 mm in length, well sclerotized, dorso-ventrally nearly symmetric; median lobe in lateral view C-shaped, narrowed at base, apically thickened, apical part abruptly narrowed and pointed at apex; parameres elongate, each with two long apical setae.

**Female.** Unknown.

#### Comparative notes.

This species is morphologically similar to the Burmese *Pseudophaniasspinicornis* Inoue & Nomura and *P.tanintharyiensis* Inoue & Nomura by the male antennal modification composed of apical six antennomeres (Inoue and Nomura 2021), e.g., antennomeres 6 greatly enlarged and distinctly larger than 5 and 7. Otherwise, these species can be readily separated by the different shapes of the antennal clubs and aedeagus. *Pseudophaniasspinicornis* has coarsely punctate head dorsum and pronotum, while *P.mulun* sp. nov. has a smooth, finely punctate pronotal disc, similar to that of *P.tanintharyiensis*.

#### Distribution.

Southwestern China: Guangxi.

#### Etymology.

This species is named after its type locality, Mulun Nature Reserve.

### ﻿Key to males from Nanling Priority Area for Biodiversity Conservation

**Table:** 

1	Antennomeres 6 enlarged, much broader than 7 (Figs 1C, 3C); median lobe of aedeagus evenly narrowing apically (Fig. 1E) or abruptly narrowed before apex (Fig. 3E), apex pointed	**2**
–	Antennomeres 6 normal, approximately as broad as 7 (Fig. 2C); median lobe of aedeagus greatly enlarged apically, apex globular in lateral view (Fig. 2E) (Guizhou)	***Pseudophaniasleigong* sp. nov.**
2	Antennomeres 5 greatly transverse, as broad as 6 (Fig. 1C); median lobe of aedeagus at middle greatly projected ventrally, projection deeply forked apically (Fig. 1D, E) (Guangxi, Guizhou)	***Pseudophaniasfurcilobus* sp. nov.**
–	Antennomeres 5 unmodified, much narrower than 6 (Fig. 3C); median lobe of aedeagus at middle smoothly curved, lacking projection (Fig. 3D, E) (Guangxi)	***Pseudophaniasmulun* sp. nov.**

## Supplementary Material

XML Treatment for Pseudophanias furcilobus

XML Treatment for Pseudophanias leigong

XML Treatment for Pseudophanias mulun

## ﻿Additional information

### Author ORCIDs

Yong-Qin Zhang https://orcid.org/0009-0004-6112-6640

Zi-Wei Yin https://orcid.org/0000-0001-6659-9448

### Data availability

All of the data that support the findings of this study are available in the main text.
